# Elastomer–Hydrogel Systems: From Bio-Inspired Interfaces to Medical Applications

**DOI:** 10.3390/polym14091822

**Published:** 2022-04-29

**Authors:** Gokhan Demirci, Malwina J. Niedźwiedź, Nina Kantor-Malujdy, Miroslawa El Fray

**Affiliations:** Department of Polymer and Biomaterials Science, Faculty of Chemical Technology and Engineering, West Pomeranian University of Technology in Szczecin, Al. Piastów 45, 70-311 Szczecin, Poland; gokhan.demirci@zut.edu.pl (G.D.); nm50247@zut.edu.pl (M.J.N.); nina-kantor-malujdy@zut.edu.pl (N.K.-M.)

**Keywords:** elastomers, hydrogels, elastomer–hydrogel systems, injectable biomaterials, adhesive surfaces, tissue engineering

## Abstract

Novel advanced biomaterials have recently gained great attention, especially in minimally invasive surgical techniques. By applying sophisticated design and engineering methods, various elastomer–hydrogel systems (EHS) with outstanding performance have been developed in the last decades. These systems composed of elastomers and hydrogels are very attractive due to their high biocompatibility, injectability, controlled porosity and often antimicrobial properties. Moreover, their elastomeric properties and bioadhesiveness are making them suitable for soft tissue engineering. Herein, we present the advances in the current state-of-the-art design principles and strategies for strong interface formation inspired by nature (bio-inspiration), the diverse properties and applications of elastomer–hydrogel systems in different medical fields, in particular, in tissue engineering. The functionalities of these systems, including adhesive properties, injectability, antimicrobial properties and degradability, applicable to tissue engineering will be discussed in a context of future efforts towards the development of advanced biomaterials.

## 1. Introduction

The structure of biological materials has been fine-tuned over millions of years of evolution. The study of live organisms to derive new principles and technologies and then apply them to man-made materials, including polymers and combined systems such as composites and hybrids, to emulate biological functions and performance, is in a central place of biomimetics (bioinspiration). Following the principles of designing materials with defined biological properties, such as bioactivity, adhesiveness, self-healing, etc., the use of polymeric materials in medicine is one of the most important application areas in restoring health, interacting therapeutically or, ultimately, replacing diseased tissues or organs. The diversity of the chemical structures, synthesis possibilities and the monomers’ origins/sources make polymers important in the controlled release of drugs, implants for tissue reconstruction, medical devices and many other areas [1,2,3,4,5]. Naturally occurring polymers were the first to be used in medical applications around the 1960s. The most common representatives of natural polymers are proteins, polysaccharides and polynucleotides [6]. The main advantage of these natural polymers is high biocompatibility due to their chemical structure and natural origin. Nevertheless, these polymers can cause an immune response due to the possible impurities remaining after processing. Natural material fabrication typically involves decellularization and the removal of antigenicity, to obtain a material that will not cause an immune response after implantation in the human body. Therefore, synthetic materials which are obtained via precision macromolecular engineering or well-established polymerization processes often have better properties, both physical and biological, and are therefore frequently developed and used in medical applications. They can be engineered as very complex supports for tissues and organs that exhibit organized heterogeneity within multiple cell types [7].

Elastomers are a class of polymeric materials that can repeatedly and easily undergo large, reversible deformations with complete recovery [8]. They are usually composed of long-chain molecules, extremely flexible due to their ability to reconfigure themselves and dissipate an applied force. The main feature of elastomers, also called “biomedical elastomers” or “bioelastomers” is their viscoelasticity combined with biodegradation, thus making them suitable for medical applications as drug delivery systems [9,10], biosensors [11,12], artificial organs [13,14], materials for regenerative medicine [15,16], tissue engineering [17,18,19] and veterinary medicine [20]. They are suitable for medical applications due to their great attributes such as a 3D-crosslinked network structure, good mechanical properties and the possibility of tailoring degradation by introducing functional groups within the structure [18].

Hydrogels (HGs) are macromolecular structures consisting of polymer networks with the ability to absorb water without any dissolution [21]. Water is held between the polymer chains, which gives the material elastic features; however, hydrogels become a rigid macromolecular tangled structure when the water evaporates. The features of HGs such as their hydrophilic character, a similar structure to tissues, ability to degrade, chemical stability and the ability to absorb an immense amount of water or biological fluids, make HGs eligible for tissue engineering and regenerative medicine (TERM) [22].

Synthetic materials were designed as structural components of medical devices with tailored stability/degradation time. However, rapid development of materials engineering has triggered the new trend to incorporate the natural polymers’ sophistication into the synthetic polymer structures, to introduce biological cues that are necessary to support or replace the targeted tissue or organ and to better understand how these features can be created more effectively [23]. Advances in manufacturing strategies have ensured additional contributions to biomaterial design [24]. Thus, combined systems such as elastomer–hydrogel systems, often inspired by nature, containing natural and synthetic structural elements/polymers of distinct but often complementary or synergistic properties, are rapidly being developed to better optimize the final performance of medical devices.

This review is focused on recent advancements in the development of elastomer–hydrogel systems derived from distinctly different polymeric materials (natural origin and synthetic, hydrophobic and hydrophilic, elastic and stiff) to create materials with functionalities and properties resembling biological tissues. Insights into bio-inspired strategies for strong interface formation between elastomers and hydrogels will be provided and their functionality, including bioadhesiveness, injectability, porosity, biodegradation and elasticity will be discussed.

## 2. Preparation of Elastomer–Hydrogel Systems

**Figure 1:**
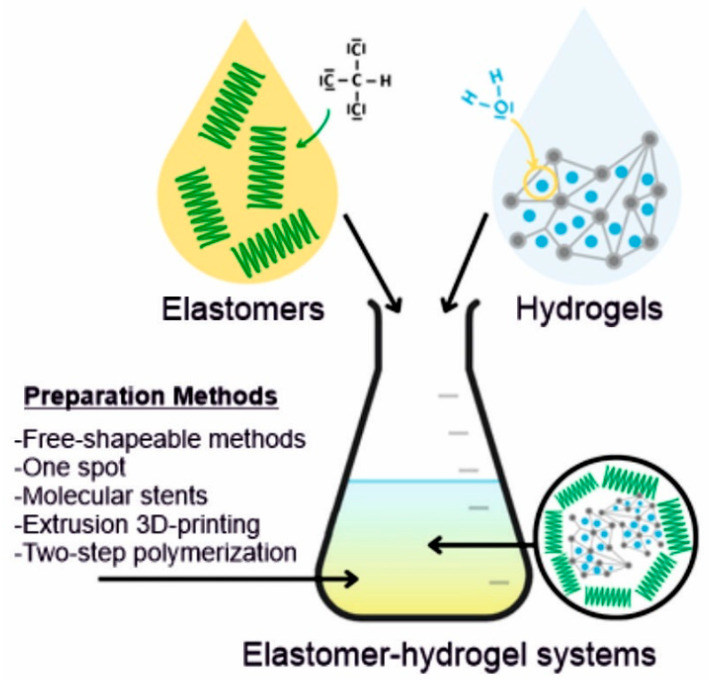
Schematic representation of elastomer–hydrogel systems. Hyphen (-) signs represent free electron pairs.

Elastomer–hydrogel systems (EHS) are the combination of two or more polymeric materials, commonly of natural and synthetic origin, offering remarkable properties and multifunctionalities through the combination of different structural components (Figure 1). These may be systems where a hydrogel is encapsulated by an elastomer matrix to prevent its dehydration or both components are forming interpenetrating polymer networks (IPN) which can be bonded to each other by covalent bonds or non-covalent interactions such as hydrogen bonding, van der Waals and electrostatic interactions.

A wide range of EHS preparation methods such as a two-step polymerization, molecular stent, one-spot, extrusion 3D-printing and free-shapeable methods results in diverse properties of the systems, thus widening their applicability in different areas [25]. In the last decades, EHS have triggered more attention due to their specific physicochemical key properties such as enhanced mildness, solubilization, density, permeability, stiffness, low surface tension, stability, mesh size and structure. Moreover, their biocompatibility, biodegradability, non-immune response and structural similarity to the extracellular matrix (ECM) have attracted the researchers to focus on new developments in medicine. The properties of EHS can be structured through selection of their chemical composition, cross-linking strategy, structure stabilization, hydrophobicity/hydrophilicity ratio, etc.

Among various groups of polymers, thermoplastic elastomers such as polyurethanes [26], poly(ε-caprolactone) copolyesters [27], poly(ether ester)s [28] and thermoset elastomers such as crosslinked polyesters [29] have been developed for heart valves and muscle applications, skin, cartilage implants, blood vessels, vascular catheter and wound dressings [30,31,32,33,34]. Simultaneously, hydrogels which show a physicochemical similarity with ECM and provide high-water content are considered as highly biocompatible materials. Therefore, the use of elastomers and hydrogels is increasing rapidly in medical applications [35,36,37,38]. The rational design of elastomers and hydrogels could be a solution to obtain highly functional elastomer–hydrogel systems with tailor-made elasticity and wettability while preserving or creating strong adhesion between the components or with the biological tissues.

**Figure 2:**
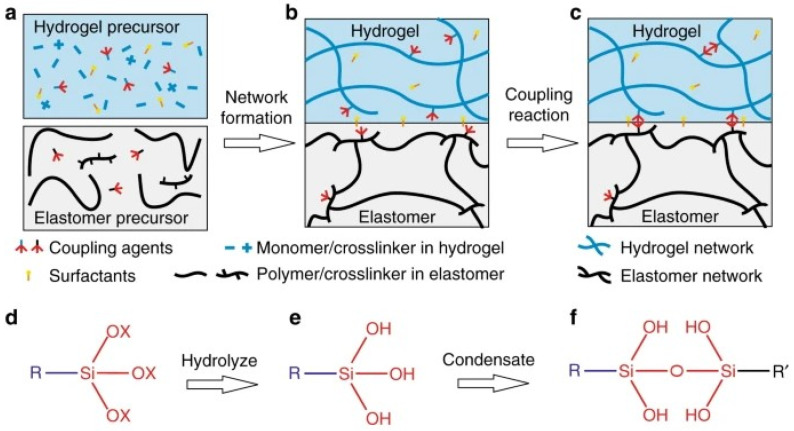
An elastomer and a hydrogel forming covalent bonds after a manufacturing process. (**a**) Silane coupling agents are mixed into the precursors of a hydrogel and an elastomer separately. (**b**) During the formation of the two networks, the coupling agents are covalently incorporated into the networks, but do not condensate. (**c**) After a manufacturing process, the coupling agents condensate, add crosslinks in the individual networks, and form bonds between the networks. A surfactant may further promote adhesion. (**d**) Silane coupling agents hydrolyze and form (**e**) silanol groups, which condensate to form (**f**) siloxane bond. Reproduced from [39] with permission. Copyright 2018 Qihan Liu et al.

Such systems can be created by physical interactions (topological entanglements) and/or chemical bonds to create a strong interlinks between elastomer and hydrogel. Different chemicals such as silane coupling agents [39], cyanoacrylates [40] and benzophenone [41], have been used to create chemical bonds between distinctly different polymers; for instance, a silane coupling agent (SCA) introduced into the precursors of a cured polydimethylsiloxane (PDMS) elastomer and polyacrylamide (PAAm) hydrogel. After manufacturing, SCA condenses and creates bonds across the interface and improves adhesion. Bonding energy differences were investigated on various addition sequences such as cured hydrogel and cured elastomer, cured hydrogel and uncured elastomer, uncured hydrogel and cured elastomer (Figure 2) [39]. Furthermore, there are techniques of combining these distinctly different components inspired by nature and it is proven that these strategies can be applied for the biomimetic devices and machines with a wide selection of elastomers and hydrogels.

## 3. Bio-Inspiration for Strong Interface Formation between Elastomers and Hydrogels

**Figure 3:**
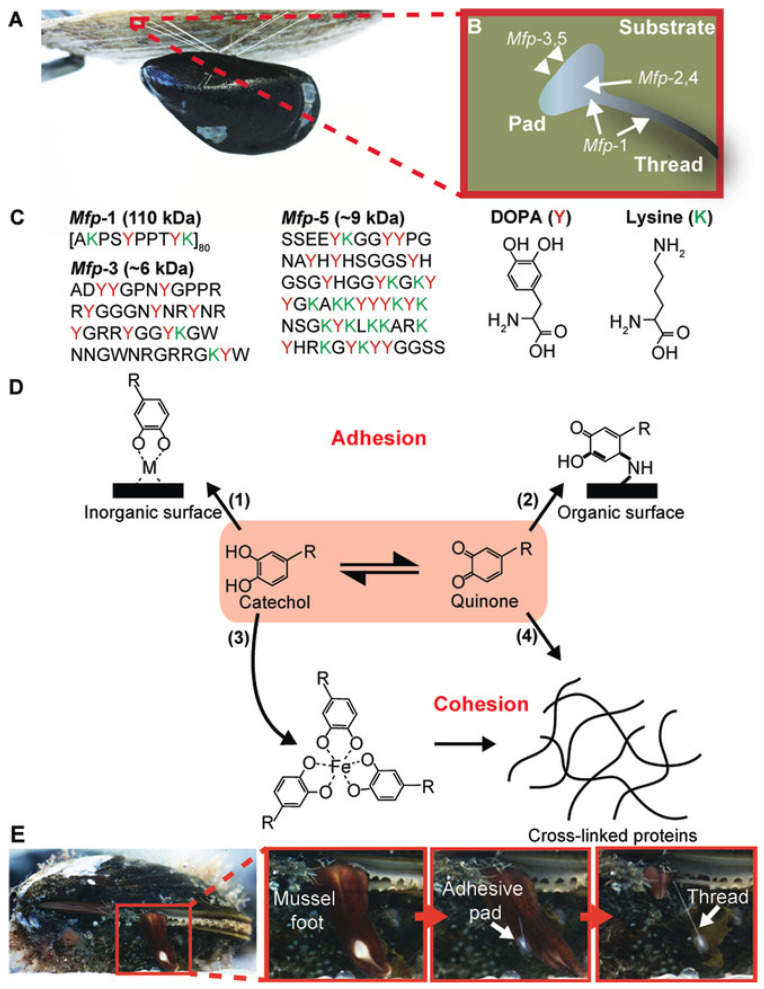
(**A**) Blue mussel attached to a solid substrate (another mussel shell) using its byssal threads. Photo credit MSc Simon Frølich (Aarhus University). (**B**) Biodistribution of mussel foot proteins (mfps) in the byssal thread and pad. (**C**) Primary amino acid sequence of mfp-1, mfp-3 and mfp-5 (Y: DOPA, K: lysine). (**D**) Scheme showing examples of the adhesive and cohesive properties of catechol-containing proteins, R represents the remainder of the mfps. (**E**) Time-lapse photography showing the molding of a byssal thread (molding time ≈ 5 min). Reproduced from [47] with permission. Copyright 2016 WILEY-VCH Verlag GmbH & Co. KGaA, Weinheim.

Many researchers are inspired by nature to create bonds between different surfaces. For instance, synthetic adhesives bio-inspired from marine organisms such as mussels, gained great attraction due to their suitability for saline and watery environments, as well as their high adhesive strength [42,43,44]. These adhesives show exceptionally high bonding strengths with various substrates, and due to research progress in the isolation and characterization of mussels’ main adhesive components, their use in medical applications such as dental and surgical glues is envisioned [45,46]. The main adhesive compound in mussels is 3,4-dihydroxyphenylalanine (DOPA) which contains catechol units to create covalent and noncovalent interactions to many different surfaces, see Figure 3 [47].

Another inspiration is taken from the ability of geckos’ feet to adhere to tough surfaces. It has inspired researchers to fabricate tissue adhesives by mimicking its nano-scaled fibrillar array structure on the bottom of geckos’ feet. Those fibrillar arrays maximize the interfacial adhesion to surfaces by capillary forces and van der Waals interactions [48,49]. However, the created adhesion is not permanent, especially to wet surfaces. Therefore, Mahdavi et.al. have developed a gecko-inspired biodegradable tissue adhesive consisting of oxidized-dextran aldehydes (DXTA) coated on nano-patterned poly(glycerol sebacate acrylate) (PGSA) produced by photolithography and reactive ion etching for medical therapies. The adhesion results showed that the fabricated DXTA-PGSA hybrid showed 2-fold higher adhesion than PGSA without DXTA [50].

These examples clearly demonstrate that nature offers a great model for the development of strong interfaces between different surfaces/materials/systems.

## 4. Components of Elastomer–Hydrogel Systems by Their Origin

**Figure 4:**
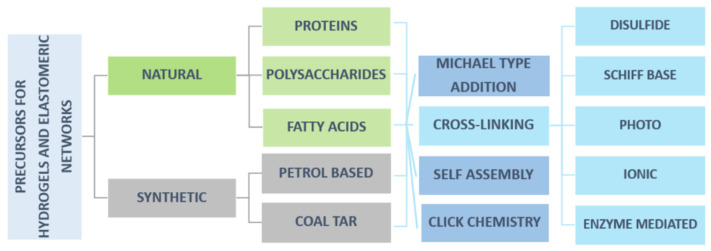
Natural and synthetic precursors of elastomers and hydrogels by their origin, reaction type and crosslinking method.

The creation of EHS involves various polymerization and crosslinking methods, with a very broad component selection for the targeted properties and application. The components of EHS can be extremely diverse and derived either from natural or synthetic sources (Figure 4). In this review, we will focus on components of EHS by the nature of their origin, including natural, semisynthetic, and synthetic raw materials.

### 4.1. Elastomers

The versatility of elastomers originates from a wide design window, where natural polymers (biopolymers) and synthetic elastomers can be used to gain the required properties for targeted applications. In this section, we have introduced the elastomers according to their source, and mechanical properties.

#### 4.1.1. Natural Elastomers

Natural elastomers are characterized by their inherent biocompatibility, biodegradation, hydrophilic character and high bioactivity, which are promising for regenerative medicine. Mainly, natural-origin elastomers are derived from proteins, including collagen, elastin, fibrin, chitin [51] and resilin [52,53]. Mechanical properties of the most popular ones (collagen, elastin and fibrin) are shown in Table 1.

The most popular elastomer is collagen, a type of fibrous protein, which is the main structural component of connective and bone tissue [56]. Collagen’s structure is mostly dominated by rigid secondary regions of triple-helix, thus resulting in 29 types of collagen. Collagen is a flexible macromolecule, with strain at break of 10–20% and a resilience of 90% [57]. The elasticity of collagen may differ depending on the structural arrangement of fibrous tracts. For instance, bone collagen has a higher density of intermolecular crosslinks than soft tissue collagen. Other important elastomers found in tissues are elastin and fibronectin [58]. Elastin mostly contains amorphous, random-coil domains which result in high elasticity of this protein, thus directly contributing to organ elasticity [59]. Interestingly, it has rubber-like properties that provide high elasticity, extensibility and resilience. Elastin can form an insoluble network as a result of hydroxylation and crosslinking. Elastin is widely used in soft tissue engineering, e.g., split-skin autografts for burn wounds, gastrointestinal patches, heart valve replacement and vascular grafts. However, natural elastin is not used often in cardiovascular prosthetic implants due to its purification, batch-to-batch variations, and high propensity to calcification due to its poorly defined purification [15]. The exceptional properties of another biomacromolecule, fibrin, are directly connected with the blood clotting process assisting wound healing. Therefore, this elastomeric protein is used in materials for skin grafts. Fibrin shows mitogenic, chemotactic and proangiogenic activities and even degradation (coagulation and fibrinolysis) products are activators of wound repair [58]. This protein is widely used in many applications due to rich bioactivity and easy manipulation. Fibrin can be a perfect candidate for the cell instructive scaffolds due to its three-dimensional organization and injectability which minimizes the invasiveness of the procedure and biodegradation within a short period of time (1–26 weeks) [60].

#### 4.1.2. Synthetic Elastomers

**Figure 5:**
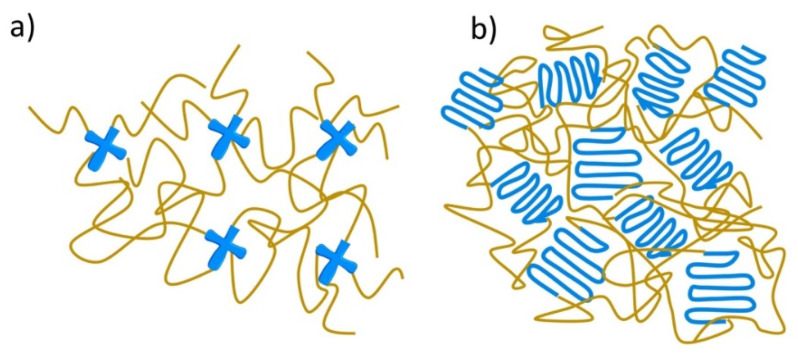
Schematic structure of chemically crosslinked (**a**) and physically crosslinked (**b**) elastomers.

In order to mimic the elastomeric and biological properties of natural tissues and organs, synthetic materials have been rapidly developing in the last decades. Their advantage over natural-based elastomers is the much wider availability of monomeric units and the synthesis approaches. The most popular synthetic elastomers belong to the group of polyesters, where the mechanical properties (elasticity) and degradation in various environments can be tailored by applying various monomers and synthesis routes, preferably in bulk, without solvents [61]. Synthetic elastomers are usually linear polymers or block copolymers formed by the polyaddition, ring-opening polymerization and polyaddition of difunctional or cyclic monomers [62,63,64,65]. The elastomer structure can be stabilized by chemical or physical crosslinking (Figure 5). Chemically crosslinked elastomer chains and/or segments are interconnected into a three-dimensional network structure by covalent bonds, which are introduced during the curing process (Figure 5a). Curing can be performed by radiation or thermal processes. The most popular elastomers in this group are poly(ethylene glycol) (PEG), poly(glycerol sebacate) (PGS) and poly(glycerol sebacate-*co*-acrylate) (PGSA). In physically crosslinked elastomers (Figure 5b), elastomeric chains/segments are associated by weak hydrogen bonds, van der Waals forces, dipolar forces, microcrystalline or glassy domains [66]. Main groups of physically crosslinked materials are segmented polyurethanes (PUs), block copolyesters and styrene-based triblock copolymers [67].

Thermoplastic polyesters and their copolymers are currently dominating the field of biomedical materials. Most of them are highly biodegradable and hydrolysable to metabolic products. The most popular ones are poly(lactic acid) (PLA), poly(glycolic acid) (PGA), poly(ε-caprolactone) (PCL) and their copolymers and blends. Polyesters can result in a direct reaction between carboxylic acid (-COOH) and a hydroxyl group (-OH), usually from alcohol. However, the synthesis route can include single or double ester exchange, acidolysis, activated condensation or carboxylate polymerization reaction with the use of acid halides [68].

An interesting example of condensation copolyesters are segmented block or random copolymers containing alcohols or acids derived from long chain fatty acids which are endogenous to the human body [65]. Many studies have proven that polyesters containing either terephthalic acid (e.g., ethylene terephthalate as in PET) or poly(butylene succinate) (PBS) are biocompatible and exhibit good mechanical properties for soft tissue-engineering applications. A common feature of biomedical thermoplastic elastomers is heterogeneous degradation as a result of which, a loss of structural integrity occurs and therefore they can be used as soft micro/nanoparticles for drug delivery [69]. In heterogeneous degradation, the crystalline domains are more resistant to degradation than amorphous regions [70,71] thus different amounts of co-monomeric units can be used as simple design tools for materials of variable degradation time.

Other recently developed elastomers are poly(diol citrate)s (PCC) synthesized through the polycondensation of citric acid (CA) and various diols which have a significant influence on the properties of the final materials. CA is one of the products of human metabolism, created during the Krebs and citric cycle [72,73]. According to Yajing Zhou et al., the molar ratio of monomers and thermosetting conditions have crucial effects on the properties of the PCC, as demonstrated by elongation ranging from 70% to 260% [72].

The polycondensation of glycerol and sebacic acid has resulted in poly(glycerol sebacate) (PGS), a highly elastomeric polymer mimicking collagen and elastin’s mechanical properties found in the extracellular matrix [74]. The inherent components of this polymer are natural metabolic compounds, where glycerol is involved in the synthesis of phospholipids and sebacic acid is important for the synthesis of fatty acid. PGS has been found to be biodegradable at low crosslink density [75]. PGS, as with many condensation polymers whose properties can be simply tailored by weight content of the monomers on the feed, is capable of forming a variety of polyester networks using low molecular weight multifunctional alcohols and carboxylic acids. PGS has extraordinary potential for soft and hard tissue engineering [76,77,78,79].

Another group of important synthetic elastomers are segmented polyurethanes. For instance, dopant-free conductive polyurethane elastomer (DCPU) was synthesized with the use of poly(caprolactone) (PCL) (biodegradable segment), aniline trimer with two amine end groups (conductive segment), and dimethylolpropionic acid (DMPA) (dopant molecule) with 1,6-hexamethylene diisocyanate (HDI) as the hard segment component. According to Cancan Xu et al., DCPUs are biodegradable; degradation can occur through hydrolysis and oxidation by enzymes. Furthermore, DCPU films and products of their degradation during cytotoxicity test with 3T3 fibroblasts showed good cell viability. The mechanical properties demonstrated high elasticity with the breaking strain ranging from 685 to 825% thus indicating the potential of this material in medical applications [26]. The mechanical properties of the commonly used synthetic elastomers are summarized in Table 2.

Recently, fatty acid-derived copolyesters have also been gaining great attention in regenerative medicine due to their decomposition/disintegration into natural components such as glycolic acid, citric acid, ricinoleic acid, etc. [86]. For instance, Ickowicz et al. synthesized copolyesters based on castor oil and citric acid by polycondensation reactions for use in soft tissue augmentation. Degradation studies showed that the branched copolyester showed less than 10% of weight loss in 30 days. The in vivo biocompatibility study was performed in rats, which revealed 20% weight loss of formulation in 9 months after post-subcutaneous administration [87]. The advantages and disadvantages of the selected natural and synthetic elastomers are presented in Table 3.

### 4.2. Hydrogels

Most hydrogels consists of natural and synthetic moieties. An important aspect is designing such a material is to properly combine the expected features of the various components for subsequent applications. Attention should be paid to chemical modifications or filling HGs with components such as drugs or cells, which may be an obstacle in combining an already processed matrix. The form of HGs to be placed in the body is usually challenging, so nowadays, the design of injectable materials has gained popularity. Great emphasis is now put on insulin delivery, which is widely described in [92]. Wound healing in hyperglycemia is difficult, therefore Wang et al. group created an antibacterial hydrogel dressing with deferoxamine. They obtained HG with good mechanical properties and self-healing properties and biocompatibility [93]. Injectable composite thermoactive hydrogels are used in bone regeneration assessment in bone tumor regeneration. Depending on the patient’s body temperature, β-sodium glycerophosphate and carbon particle hydrogels show sol–gel phase transitions and therefore irregular bone defects are built up [94]. Likewise, injectable hydrogels are typically used to prevent bleeding, for example after an arterial rupture. The solution to this problem is a hemostatic nanoporous hydrogel, combining the reaction of the transglutaminase enzyme and Schiff’s base reaction. The minimally invasive method was applied with a gelation time of about 10 s and with no need to use hemostatic clamps at both ends of the vessel damage [95]. As a positive feature, the environment of the hydrated structure of HG protects drugs and cells, and ensures good transport of nutrients to the cells [96]. Another important aspect is that all components of HGs (monomer, initiator and crosslinker) are entirely reacted during the formation process, or the side products are efficiently removed since unreacted products can significantly deteriorate chemical and physical properties. Therefore, relatively low toxic and high effective components are used [97].

HGs can be divided by their sources into natural, synthetic and semisynthetic origins. The advantage of natural components is their non-toxicity, biocompatibility and degradability. However, they are often unstable. On the other hand, synthetic HGs ensure stability but are hard to degrade. Therefore, by the combination of natural and synthetic components, the final properties can be easily tuned for medical applications [98,99,100].

#### 4.2.1. Natural Hydrogels

HGs occurring in nature can be divided into two main groups: polysaccharides, such as chitosan, hyaluronan, alginate, agarose, and proteins. Natural HGs promote good cell interactions and adhesion thanks to their origin. As natural materials, they are also marked by their biodegradability, biocompatibility and low cytotoxicity. Based on polysaccharides that connect using the Schiff-type reactions, aldehyde hydroxyethyl starch (AHES) and amino carboxymethyl chitosan (ACC), the rapidly forming in situ hydrogel has a homeostatic ability, which is a particularly attractive property for tissue adhesives [101]. Natural hydrogels are mainly used for articular cartilage tissue engineering due to their similar constituent of water which is from 60 to 90% for hydrogel and about 70% in the ECM of cartilage tissue. Stimuli-responsive polysaccharide hydrogels are intelligent hydrogels that change form in response to factors such as pH, light, pressure, etc. Polysaccharide HGs exhibit storage properties to immobilize molecules, which makes them interesting for biomedical usage [102].

#### 4.2.2. Synthetic and Semisynthetic Hydrogels

Synthetic hydrogels can be generated chemically by crosslinking polymers with radiation, click chemistry reactions [103,104], or Michael type addition [105], and are called chemical HGs. Physical HGs can be formed by warming or cooling the polymer solution, mixing polyanion and polycation solutions, or lowering the pH to attach via hydrogen bond polymers in an aqueous solution [97,106,107,108]. Both chemical and physical HGs are inhomogeneous due to their interior structure. They consist of areas entitled “clusters” that swell a low amount of water because of high crosslinking density. In contrast, there are also low crosslinked density regions or even filled water spaces or macropores. Synthetic HGs offer an advantage over natural HGs, in that they are more controllable, but less biologically active. The composition or architecture of the matrix needs to be considered. Features of synthetic and semisynthetic hydrogels such as biocompatibility, reproducibility, mechanical properties and biodegradability are crucial. Therefore, attention goes towards the selection of HGs components for specific applications.

In designing a synthetic hydrogel for application as a carrier of cells, one should consider the balance that needs to be kept between the biodegradation time and mechanical properties of HGs and ECM, and the growth of the desired tissue. Polymeric particles (macromolecules and proteins), similar to natural ones, influence the structure of tissues, and behavior of cells, which contributes to the regulation of cell functions. Some of the biodegradable synthetic HGs are poly(N-isopropylacrylamide) (PNIPAAm), poly(ethylene oxide)-poly(propylene oxide)-poly(ethylene oxide) (PEO-PPO-PEO), triblock copolymer consisting of poly(lactic acid-*co*-glycolic acid) (PLGA) and poly(ethylene glycol) (PEG) (PLGA-PEG-PLGA) or poly(ethylene glycol)–polylactide-poly(ethylene glycol) (PEG-PLA-PEG). PEG derivatives which are mostly combined with fibrinogen, hyaluronic acid or poly(propylene fumarate) (PPF) are used in particular adhesives and scaffolds [106,107]. Zant and Grijpma synthesized and crosslinked macromers based on poly(trimethylene carbonate) (PTMC), poly(D,L-lactide) (PLL), poly(ε-caprolactone) (PCL) and PEG by using photopolymerization, and the obtained synthetic HGs showed high water uptake, remarkable ability to promote cell adhesion and proliferation [109]. As another example, modified alginates with 2-aminoethyl methacrylate (AEMA) subjected to photo-crosslinking are excellent materials for creating a filler or a scaffold because of slow degradation. Gelation occurred in vivo, and the mechanical properties were improved, supporting the components of cartilage tissue [99]. For instance, acrylamide-based hydrogels filled with MgO controlled the initial burst release through modification of the structure of the matrix, acting as nanosized drug reservoirs [110]. Additionally, acrylamide-based vaginal rings are designed to deliver acyclovir into the vagina, thus increasing protection against infection after childbirth. This is a novel approach to cross-linked hydrogels which gradually release drugs in this scope [111]. The mechanical properties of commonly used hydrogels are given in Table 4.

The main disadvantage of synthetic hydrogels is the lack of biologically recognized cell attachment sites and thus, poor cell proliferation. Despite the promising mechanical properties, the proliferation of cells proves the usefulness of the hydrogel. Semisynthetic hydrogels make it possible to combine the desired properties. Due to this combination, semisynthetic hydrogels have the appropriate physical properties and are reproducible but also have the desired biological properties, which result in a wider spectrum of utility [7]. For instance, a synthetic hydrogel consisting of sodium p-styrene sulfonate (NaSS) and N,N-dimethyl acrylamide (DMAAm), which was negatively charged NaSS and neutral DMAAm, on which there is no proliferation. This combination was examined for the adhesion, migration and proliferation of cells. In conclusion, even if the cell behavior was satisfactory, they were unable to proliferate [119]. In contrast, hydrogels made from poly(ethylene glycol) (PEG) and proteins such as fibrinogen, gelatin and albumin were able to support the neurite extension and glial cell migration from the dorsal root ganglion, in contrast to the control PEG hydrogel [120].

## 5. Biofunctionalities of Elastomer–Hydrogel Systems

**Figure 6:**
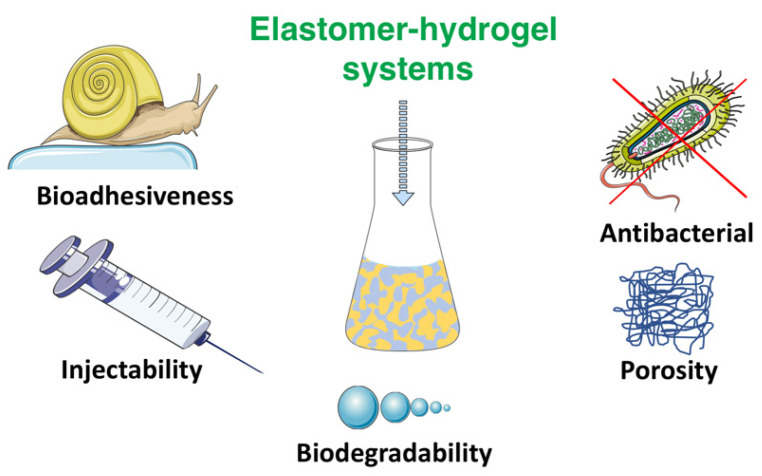
Functions and performance of elastomer–hydrogel systems.

EHS are gaining increased interest for medical applications due to their unique combination of properties, often emulating live organisms’ function and performance. Some of the sophisticated properties found in biomimetic materials will be discussed with emphasis on bioadhesiveness, injectability, antibacterial properties, biodegradability and porosity which are important for tissue engineering (Figure 6).

### 5.1. Bioadhesiveness

**Figure 7:**
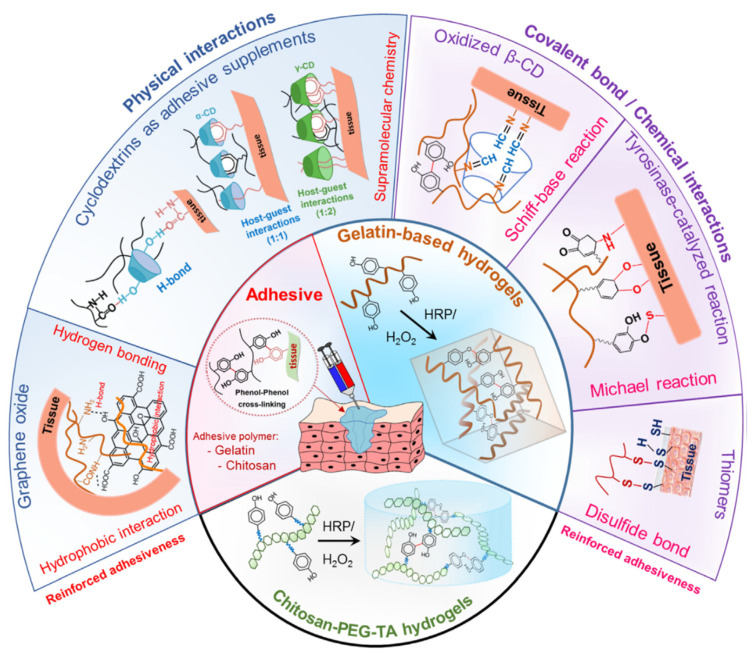
Development strategy of in situ forming horseradish peroxidase (HRP) catalyzed hydrogels with significantly enhanced adhesiveness through polymer choices and additional crosslinking. Reproduced from [48] with permission. Copyright 2019 The Korean Society of Industrial and Engineering Chemistry. Published by Elsevier B.V. All rights reserved.

Most of the medical applications, especially surgical procedures, require tissue adhesives, sealants, and hemostatic agents. Those bioadhesives are mostly a glue to bind the tissues, seal the gaps or cracks and initiate the formation of blood clots, respectively [121,122]. Synthetic compounds which show adhesive properties such as poly(ethylene glycol) diacrylate (PEGDA) [123], N,N-dimethylaminoethyl methacrylate-co-methyl methacrylate (NDMEM) [124], gelatin methacrylate (GelMA) [125], tannic acid (TA) [126], etc., have been successfully introduced by physical or chemical processes into the patches or scaffolds with the development of materials science. This approach has gained successful outcomes in medical applications thanks to the adhesion ability of those materials to various tissues such as soft tissue, bone and skin [127,128,129,130]. However, their lack of robust and reversible adhesion abilities limit their application efficiencies. Therefore, inspiration from nature provides enormous information on how to develop materials with versatile adhesion capacities for both wet and dry surfaces. Determination of the key compounds within the various species has opened the way to introducing these compounds into the structured materials for medical applications. Thanks to these compounds, EHS can act fully or partially as bioadhesives, depending on the functional groups introduced that are inspired by nature (Figure 7). EHS can be structured to achieve the desired, controllable and reusable adhesion strength in wet environments.

Recently, bioinspired adhesives have attracted great attention due the combination of natural functionality realized through synthetic approaches. For instance, mussels show extremely good adhesion with high binding strength to various surfaces under wet conditions [131,132,133]. It was found that the catechol unit is the main factor that allows mussels to adhere to a variety of surfaces [134,135]. Materials containing catechol units can be used to create covalent and non-covalent attachments to various substrates for many medical applications, including drug delivery systems and wound healing [47,136,137].

### 5.2. Injectability

**Figure 8:**
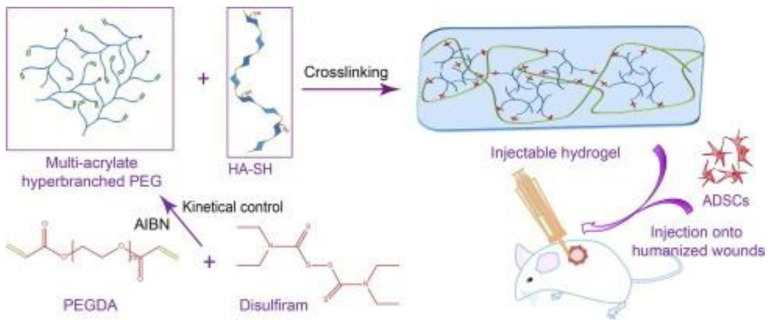
Schematic presentation of encapsulated adipose-derived stem cells (ADSCs) by an injectable hybrid hydrogel. Reproduced from [143] with permission. Copyright 2018 Acta Materialia Inc. Published by Elsevier Ltd. All rights reserved.

**Figure 9:**
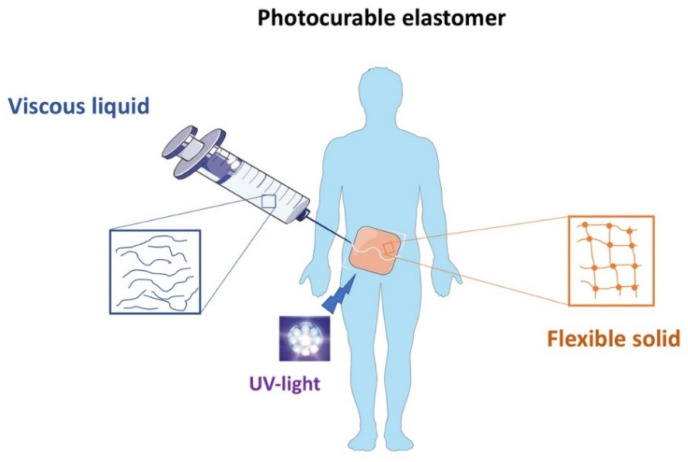
Schematic of injectable photocurable elastomer patch for small hernia repair.

Traditional surgeries are increasingly being replaced by less invasive methods that shorten an overall procedure and the patient’s recovery time. Especially, in tissue engineering, the focus is on to improving the materials’ performance by their injectability [138]. The injectable systems can efficiently deliver particles such as drugs (antibiotics, anesthetics), biomolecules (fibrin), fillers (silica nanoparticles) or genes (DNA, siRNA) [139]. An attractive model, developed by Li et al., is an injectable probe for measuring oxygen in tissues [140]. Hydrogels containing N-isopropylacrylamide copolymer macromers for mesenchymal stem cell (MSC) delivery allow the formation of bone bridges, promoting the viability of MSCs, and can be used to create hard tissues, due to gelatin microparticles (GMP) which are enzymatically digestible porogens and sites for cell attachment [141]. Another long-term persistent hydrogel is the photo-crosslinked material composed of a double-network of modified sodium alginate and gelatin created by the Schiff base reaction [142]. Collectively, different works have clearly demonstrated the huge potential of injectable materials for biomedical applications. Xu et al., produced an injectable EHS consisting of hyperbranched multi-acrylated poly(ethylene glycol) macromers (HP-PEGs) and thiolated hyaluronic acid (HA-SH) and used it as a stem cell delivery system for diabetic wound healing (Figure 8) [143]. It is also worth noting that new injectable and photocurable elastomers containing fatty acid derivatives can be successfully used for minimally invasive surgical protocols in the repair of small hernia defects (Figure 9) [144].

### 5.3. Biodegradation

Biodegradable materials are now tending to become the most commonly used materials in medical applications due to their gradual bio-resorption into the human body [145]. Biodegradability is one of the key properties for the materials which are used in medical applications. It should be considered that the degradation rate must be consistent with the healing and regeneration process. Various crosslink densities, crosslinking mechanisms and component types were applied to control the degradation rate of such systems [146,147]. The most commonly used biodegradable materials consist of homo- or copolymers of alpha-hydroxy acids, such as lactic and/or glycolic acids.

**Figure 10:**
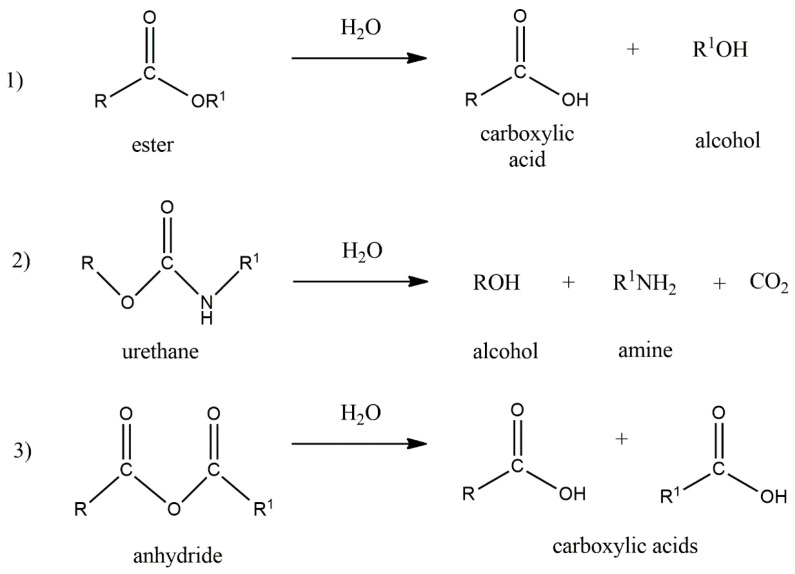
Hydrolytic degradation of (**1**) ester, (**2**) urethane, (**3**) anhydride groups occurring in elastomers.

Biodegradation can be triggered either by water (hydrolytic degradation) and/or enzymes (enzymatic degradation) within the body. The chemical structure of a polymer has the greatest influence on the type of degradation. Other important factors are chemical composition, the type of crosslinking bonds, molecular weight and its distribution, porosity, stereochemistry and chain mobility [148]. The elastomeric part of the EHS usually tends towards hydrolytic biodegradation due to its molecular chain structures sensitive to water (Figure 10). The hydrolysis of ester bonds usually leads to the creation of carboxyl and hydroxyl end groups, whereas natural biomaterials tend to degrade enzymatically.

The injected and/or implanted EHS can be degraded by oxidative (catalases, horseradish peroxidase and xanthine oxidase) or hydrolytic (protease, hydrolase, phosphatases, lipase and esterase) enzymes when exposed to body fluids and tissues [149,150,151]. Inflammatory cells (e.g., macrophages and leukocytes) create reactive oxygen species such as hydrogen peroxide, superoxide and nitric oxide during the inflammatory response to foreign materials [152]. EHS can be cut up by those species which are contributing to material degradation whereas the hydrolytic enzymes hydrolyze the components of the hybrid network to help in the absorption of nutrients and solutes.

For instance, a poly(caprolactone) (PCL)/gelatin(Gel) scaffold (sublayer) was electrospun on a dense polyurethane (PU)/propolis(EEP) (top layer) membrane to fabricate a bilayer wound dressing. It was demonstrated that the EHS combining a synthetic polymer with a natural one could enhance the stability of the scaffold. Hydrolytic and enzymatic degradation studies showed that PU/EEP membrane exhibited a slower degradation rate in comparison with a PCL/Gel hybrid structure. In the case of hydrolytic degradation, the total mass loss after 28 days for PU/EEP and PCL/Gel was found to be 1.9 and 76%, respectively [153].

### 5.4. Porosity

The porosity is an important feature in medical applications, especially in scaffolds [154,155]. The pore architecture and interconnectivity have a beneficial role in proliferation, cell survival and migration to create functional materials, and secrete ECM. Therefore, scaffold porosity is a must for homogenous cell distribution and interconnection throughout engineered tissues [156,157]. Additionally, pore size can have an effect on the cell growth, vascularization, nutrients and oxygen diffusion, especially in the absence of a functional vascular system [133,134,135,158,159,160]. Various techniques, components and ratios are used to obtain controlled pore size and architecture scaffolds. For instance, Kanimozhi et al. prepared a chitosan/poly(vinyl alcohol)/carboxymethyl cellulose (CP-CMC) porous scaffold by simple freeze drying and salt leaching techniques. Among scaffolds, 1:1 weight ratios showed significantly high porosity as compared to other ratios. The incorporation of CMC enhanced the scaffold porosity from 50 to 90% by increasing the molar ratio of CMC. However, when comparing the freeze-dried scaffolds and salt-leached scaffolds of 1:1 weight ratio, the 50% CP:50% CMC material showed a higher porosity of 90% in salt-leached and 70% in freeze-dried scaffolds, respectively. The reason was explained thus: with the increase of CMC ratio, the actual volume occupied by the molecules decreased [161].

In another study, Morris et al. produced porous elastomer–hydrogel scaffolds of chitosan/polyethylene glycol diacrylate (CS/PEGDA) using 3D bioprinting by a stereolithography method to create internal pore and macroscopic shapes. They achieved varied pore sizes by changing the CS molecular weight ratios. For instance, the average pore size of the pure PEGDA scaffolds increased from 24% to 67% by the addition of low molecular weight CS (LMWCS) (MW = 50–190 kDa) into the scaffold with the ratio LMWCS:PEGDA at 1:7.5. These kinds of studies show that controlled pore size and architecture can be achievable for specific needs in medical applications [162].

### 5.5. Antibacterial Surfaces

Antibacterial materials, especially surfaces, are playing an important role in protecting from contamination and eliminating bacteria from skin tissue and the surfaces of medical devices and implants. Bacterial adhesion is the main cause of the creation of 3D biofilm complex structures which infect the surrounding tissues. Therefore, new strategies which eliminate biofilm-based issues are applied. Hence, EHS which contain antibacterial components are being developed. For instance, Piarali et al. fabricated a fiber mesh based on the surface modification of polyhydroxyalkanoate, using an electrospinning technique, for tissue regeneration. In this study, basically an EHS was created by a synthetic antimicrobial peptide with anti-biofilm and strong bactericidal properties [149].

In another study, Muzammil et al. created elastomer–hydrogel scaffolds containing castor-oil-reinforced chitosan with various hydrophilic polymers. The obtained EHS showed antibacterial and hemostatic activities with good mechanical properties. Therefore, such systems could be good candidates for skin tissue regeneration and wound healing applications [163].

## 6. Elastomer–Hydrogel Systems for Soft Tissue Engineering Applications

The development of advanced systems for tissue engineering applications has been widely studied over the last decades. Specific interactions between the components, the combination of raw material advantages and the molecular organization of these systems dictates the direction of the tissue engineering applications. Different EHS systems which combine different classes of elastomers and hydrogels in one material with large yield formulations and many advantages, such as high interaction with targets to enhance their performance have been effectively developed.

**Figure 11:**
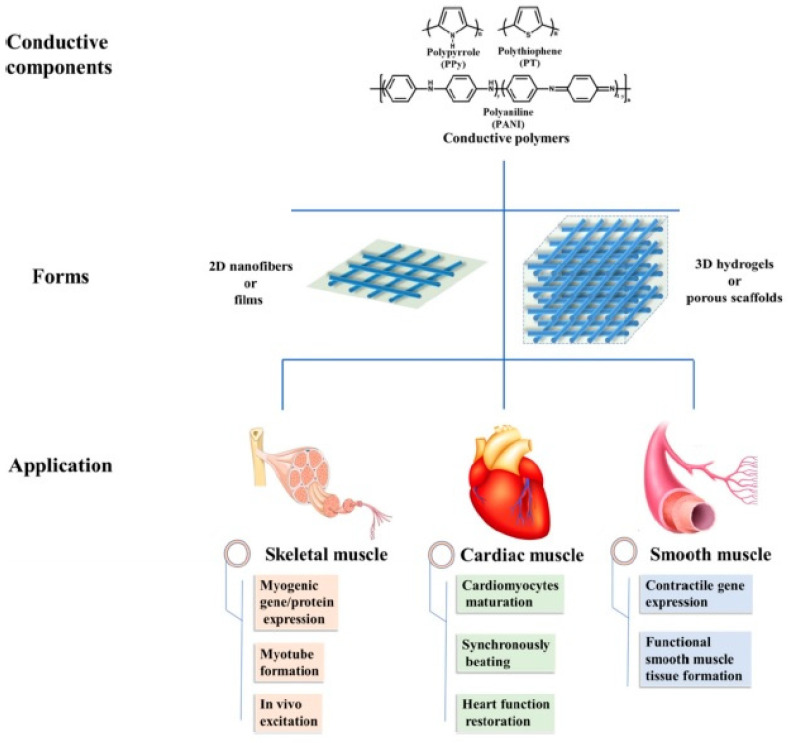
Main conductive materials, scaffold forms, and applications in muscle tissue engineering. Reproduced from [164] with permission. Copyright 2020 Omid Yousefzade et al.

EHS play an important role in the success of tissue engineering approaches, as they guide the structure of developing tissues, gaining mechanical and physical stability, and migrating cells or delivering the molecules to transplanted areas. Those highly efficient EHS find applications in soft, bone, skin, neural and cardiac tissue engineering [164,165,166,167] (Figure 11).

**Figure 12:**
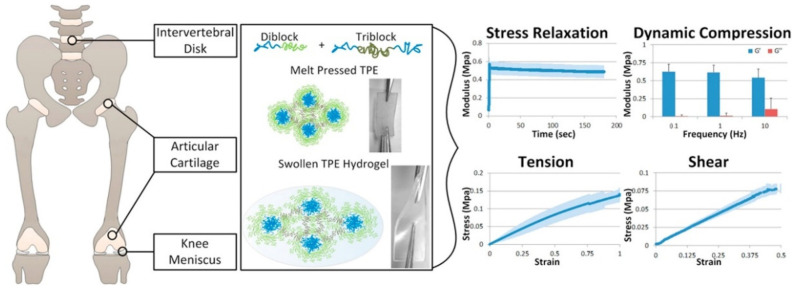
Mechanical viability of a thermoplastic elastomer hydrogel as a soft tissue replacement material. Reproduced from [168] with permission. Copyright 2018 Elsevier Ltd. All rights reserved.

For instance, Fischenich et al. has developed a thermoplastic elastomer (TPE) hydrogel system for soft tissues, especially for articular cartilage, the knee meniscus, etc. The created system was based on a blend of unreacted ω-hydroxy-polystyrene-b-poly(ethylene oxide) (SO) and coupled polystyrene-b-poly(ethylene oxide)-b-polystyrene (SOS). The obtained TPE hydrogel system could be a promising candidate for soft tissue replacement with a comparable equilibrium compressive modulus of approximately 0.5 MPa and shear modulus of 0.2 MPa (Figure 12) [168].

Lewis et al. reported a thermoplastic elastomer–hydrogel system based on the prefabrication of an efficient nanoscale network architecture using the melt-state ω-hydroxy-polystyrene-b-poly(ethylene oxide) (SO) and polystyrene-b-poly(ethylene oxide)-b-polystyrene (SOS) as amphiphilic block copolymers. They proved by physical and mechanical analysis that the obtained systems have relevant moduli and water compositions, subsecond elastic recovery rates, negligible hysteresis, and unprecedented resistance to fatigue over hundreds of thousands of compression cycles. They suggested that such hydrogels may have tremendous promise beyond the synthetic soft tissue engineering applications for which they have been targeted [169]. In another study, Remya et al. synthesized EHS by modifying PCL with different molecular ratios of water soluble polymer PEO using the electrospinning technique to create scaffolds. The weight loss for pure PCL was 8.5% whereas for PCL/PEO blends with 50:50 ratios and differing the molecular weight of the PEO (10 k g/mol vs. 60 k g/mol), the weight loss was 41.7 and 48.7%, respectively after 3 months. The study also showed that the properties of PCL scaffolds such as cell viability, mechanical properties and hydrophilicity were increased by the incorporation of PEO and these materials could be possible candidates for bone tissue engineering applications [169].

## 7. Conclusions and Perspectives

Inspiration from nature is a driving force in the development of various functional structures, including elastomer–hydrogel systems of great promise for medical applications. Recent advancements in (nano)materials science show that the rational design of hybrid systems can result in highly functional materials of good adhesion strength, injectability and the desired biological properties for specific medical applications. The variety of elastomers and hydrogels for the creation of elastomer–hydrogel systems continues to grow, and highly advanced materials are constantly being developed to fulfill all the requirements of the medical industry. Bio-inspired materials obtained from natural-resource-based elastomers, including fatty acids, have the benefits in biocompatibility and biodegradability as simultaneous optimal physicochemical and mechanical properties. In addition, natural hydrogels can overcome the potential long-term side effects of synthetic materials since biodegradation products are non-toxic and biocompatible.

In spite of the unique advantages of elastomer–hydrogel systems, the disadvantages of those systems still remain. The main disadvantages of elastomer–hydrogel systems are the weak adhesion between elastomer and hydrogel due to their nature (elastomers are hydrophobic, whereas hydrogels are hydrophilic), and increased water loss. However, those challenges can be overcome by using a nanocomposite and/or double-network hydrogel systems to improve the strength and toughness of hydrogels and thus providing better integrity of the system. In addition, the improvement of the adhesion between the components is a key question to be answered. Even though some progress has been made in the fabrication of elastomer–hydrogel systems to increase interfacial adhesion by using covalent bonding, noncovalent mechanisms and topological adhesion, further investigation in this field is needed to promote their practical applications.

Clearly, future efforts should concentrate on a better understanding of the interactions of those materials with each other and with natural body tissues, thus opening new chapters in bio-inspired materials science and engineering. Finally, such advanced bio-inspired elastomer–hydrogel systems may lead to the production of a novel class of materials on a commercial scale that are easily processable, highly biofunctional and easily applied/administrated.

## Figures and Tables

**Table 1:** Mechanical properties of selected proteins.

Proteins	Dominant Amino Acids	Distribution	Young Modulus [Mpa]	Tensile Strength [Mpa]	Elongation [%]	References
Collagen	35% Glycine, 12% Proline	Bone, teeth, vasculature, organs	100–2900	5–500	5–50	[53]
Elastin	32% Glycine, 21% Alanine	Skin, lungs, vasculature	0.3–0.6	0.36–4.4	100–220	[15]
Fibrin	45% Glycine, 30% Alanine	Blood	1.7–14.5	0.01–0.02	100	[54,55]

**Table 2:** Mechanical properties of selected synthetic elastomers.

Material	Crosslinking Type	Young Modulus [Mpa]	Tensile Strength [Mpa]	Elongation [%]	References
Poly(diol citrate) (PCC)	chemical	140–1737 *	171–977 *	70–260	[72]
Poly(glycerol sebacate) (PGS)	chemical	0.056–1.5	0.5	40–450	[77]
Dopant-free conductive polyurethane elastomer (DCPU)	chemical	0.5–3.8	9.6–20.3	170–190	[26]
Poly(glycerol sebacate-*co*-acrylate) (PGSA)	chemical	0.05–1.38	0.05–0.5	42–189	[74]
Poly(caprolactone) (PCL)	physical	210–340	10.0–60.0	300–1200	[80,81,82]
Poly(butylene succinate) (PBS)	physical	550	20.0–40.7	100–224	[83,84,85]
Poly(glycolic acid) (PGA)	physical	6900	68.9	15–20	[80]

* depending on analysis conditions (temperature, strain rate, etc.).

**Table 3:** Advantages and disadvantages of selected natural and synthetic elastomers.

Elastomer	Advantages	Disadvantages	References
Collagen	Wide range of elasticity depending on the origin of protein, can be prepared by crosslinking, show low antigenicity	hard to control degradability	[57]
Elastin	Rubber-like properties	demanding purification process, high propensity to calcification	[15,31]
Fibrin	Bioactivity (mitogenic, chemotactic and proangiogenic activities), degradation products (coagulation and fibrinolysis) are activators of wound repair	rapid degradation	[54]
Poly(lactic acid) (PLA)	Easy to print (low melting point), highly biocompatible and biodegradable	the lack of cell-recognition signals	[88]
Poly(glycerol sebacate) (PGS)	Can mimic mechanical properties of collagen and elastin, degradation product are a natural metabolic compound	the lack of cell-recognition signals	[74,77]
Poly(ε-caprolactone) (PCL)	Highly elastic, slow degradation time (1–2 years)	the lack of cell-recognition signals	[89,90]
Poly(butylene succinate) (PBS)	Controlled biodegradability,	the lack of cell-recognition signals	[91]

**Table 4:** Mechanical properties of commonly used hydrogels.

Synthetic HGs	Crosslinking Type	Young Modulus [Mpa]	Tensile Strength [Mpa]	Elongation [%]	References
Poly(ethylene glycol) (PEG)/polydimethylsiloxane (PDMS) hydrogel	chemical	0.006–0.36	0.02–0.42	30	[112]
Chitosan (CS) and poly(vinyl alcohol) (PVA) (CS/PVA)	chemical	2.3–2.5	6.0–9.70	16.3–28.1	[113]
Tunicate cellulose nanocrystals (TCNCs) aligned (anisotropic d-Gel)	physical	152.1	13.7–56.2	1400	[114]
Aluminum ion cross-linked hydrogel (Gel) high-modulus hydrogels (HM-Gel)	physical	0.59–1.94	1.26–1.74	550–650	[115]
Carboxymethyl cellulose/polyacrylic acid hydrogel (CMC/PAA)	physical	0.065–0.18	0.40–0.85	350–700	[116]
PDLLA-dMA-PCL-dMA-PEG-dMA hydrogel	physical	1.4 ± 0.2	0.47 ± 0.06	84 ± 22	[117]
Poly(trimethylene carbonate dimethacrylate) hydrogel (PTMC-dame)	physical	1.04 ± 0.04	0.46 ± 0.07	159 ± 43	[118]

## Data Availability

Not applicable.
